# The prognostic role of cardiac and inflammatory biomarkers in extubation failure in patients with COVID-19 acute respiratory distress syndrome

**DOI:** 10.1186/s13613-025-01425-3

**Published:** 2025-01-09

**Authors:** Carline N. L. Groenland, Adinde H. Siemers, Eric A. Dubois, Diederik Gommers, Leo Heunks, Evert-Jan Wils, Vivan J. M. Baggen, Henrik Endeman

**Affiliations:** 1 Department of Intensive Care, Erasmus MC, P.O. Box 2040, 3000 CA Rotterdam, The Netherlands; 2 Department of Cardiology, Thorax Center, Cardiovascular Institute, Erasmus MC, Rotterdam, The Netherlands; 3 Department of Intensive Care, Radboud University Medical Center, Nijmegen, The Netherlands; 4 Department of Intensive Care, Franciscus Gasthuis & Vlietland Ziekenhuis, Rotterdam, The Netherlands; 5 Department of Intensive Care, OLVG, Amsterdam, the Netherlands

**Keywords:** Extubation failure, Mechanical ventilation, High-sensitivity Troponin T, N-Terminal pro-B-Type natriuretic peptide, Interleukin-6, Procalcitonin, Biomarkers, COVID-19

## Abstract

**Background:**

Extubation failure is associated with an increased morbidity, emphasizing the need to identify factors to further optimize extubation practices. The role of biomarkers in the prediction of extubation failure is currently limited. The aim of this study was to investigate the prognostic value of cardiac (N-terminal pro–B-type natriuretic peptide (NT-proBNP), High-sensitivity Troponin T (Hs-TnT)) and inflammatory biomarkers (Interleukin-6 (IL-6) and Procalcitonin (PCT)) for extubation failure in patients with COVID-19 Acute Respiratory Distress Syndrome (C-ARDS).

**Materials and methods:**

In this single-center retrospective cohort study, patient characteristics and laboratory measurements were extracted from electronic medical records. Patients were eligible for inclusion if they were extubated after mechanical ventilation. The primary endpoint was extubation failure, defined as the need for reintubation or death within the next seven days after extubation, regardless of whether post-extubation respiratory support was used. Uni- and multivariable logistic regression was performed to investigate the association between biomarkers and extubation failure. Biomarkers were log_2_ transformed.

**Results:**

Of the 297 patients included, 21.5% experienced extubation failure. In univariable analysis, NT-proBNP (OR 1.24, 95% CI 1.06–1.47), Hs-TnT (OR 1.72, 95% CI 1.37–2.19) and PCT (OR 1.38, 95% CI 1.16–1.65) measured on the day of extubation were significantly associated with extubation failure. After multivariable adjustment for clinical variables (age, duration of mechanical ventilation, SOFA score), Hs-TnT was the only biomarker that was independently associated with extubation failure (adjusted OR 1.38, 95% CI 1.02–1.90). Patients with both elevated Hs-TnT (≥ 14 ng/mL) and elevated PCT (≥ 0.25 ng/mL) carried the highest risk of extubation failure (46%), while in patients with normal Hs-TnT and PCT values, only 13% experienced extubation failure.

**Conclusions:**

Hs-TnT, NT-proBNP and PCT measured on the day of extubation are associated with extubation failure in mechanically ventilated patients with C-ARDS. Since Hs-TnT is the only biomarker that is independently associated with extubation failure, Hs-TnT could offer additional objective measures for assessing readiness for extubation. Future studies should focus on an integrative approach of biomarkers combined with relevant clinical factors to predict extubation failure.

**Supplementary Information:**

The online version contains supplementary material available at 10.1186/s13613-025-01425-3.

## Introduction

Liberating patients from mechanical ventilation as soon as possible is pivotal, as prolonged mechanical ventilation is associated with increased morbidity and mortality [1, 2]. Despite a successful spontaneous breathing trial (SBT), extubation failure rates are reported up to 10–15% [3–8]. In patients with COVID-19 failure rates are even higher up to 22% [9, 10]. This is an important setback in the recovery of critically ill patients. To lower extubation failure rates without unnecessarily delaying extubation, better predictors are needed to estimate readiness for extubation. In addition, predictors can also reveal underlying causes of potential failure. Recently, the potential importance of biomarkers and their role in predicting extubation failure was highlighted [11]. Major advantages of biomarkers are that they are widely and usually rapidly available. Plasma biomarkers of cardiac and inflammatory injury can provide objective and quantifiable measures of underlying processes that are indicative of a patient’s readiness for successful extubation. Both cardiac biomarkers (High-sensitivity Troponin T (Hs-TnT)), and N-terminal pro–B-type natriuretic peptide (NT-proBNP)), and inflammatory biomarkers (Interleukin-6 (IL-6) and Procalcitonin (PCT)) have been associated with (COVID-19) disease severity and mortality [12–20]. Several studies have shown that by the down regulation of ACE II due to the SARS-CoV-2 infection, Angiotensin II is elevated which can lead to hypertension, heart failure and lung dysfunction [21–23]. Furthermore, severe inflammatory responses among which the cytokine release syndrome [24] (measured by PCT and IL-6) might act as a regulator in cardiomyocyte injury [25], and studies have described the role of inflammation in causing heart failure and myocardial infarction [26–30]. In addition, an inflammatory state in itself also increases the risk of unfavorable outcomes in patients with coronary heart disease [31].

The association between these biomarkers and extubation failure in patients with COVID-19 acute respiratory distress syndrome (C-ARDS) has not been extensively studied. An association between elevated peak cardiac troponin and extubation failure has been reported in patients with C-ARDS [32]. Furthermore, levels of IL-6 were predictive for reintubation risk in the general population of ARDS patients [18]. However, the value of biomarkers measured on the day of extubation has not yet been investigated. Elevated levels of (NT-pro)BNP have only been related to extubation failure in the general ICU population and in patients with COPD [33, 34], but not in patients with C-ARDS. The aim of this study was to investigate the association between cardiac (NT-proBNP and Hs-TnT) and inflammatory biomarkers (IL-6 and PCT) on the day of extubation and extubation failure in patients with COVID-19 acute respiratory distress syndrome (C-ARDS).

## Methods

### Study design and population

This retrospective cohort study was conducted at the Erasmus Medical Centre, a tertiary hospital in Rotterdam, the Netherlands. Mechanically ventilated adult (≥ 18 years) ICU patients with C-ARDS (defined according to the Berlin criteria [35]) as primary reason for admission between February 28th, 2020 and March 31st, 2022, were eligible for inclusion. Patients were included if they had been extubated from mechanical ventilation. Extubation was initiated when patients met ‘ready to wean’ criteria (Supplemental Table 2) and successfully completed a 30-min spontaneous breathing trial with a T-piece [4]. Exclusion criteria were terminal illness (i.e., extubation as part of discontinuation of treatment), do not reintubate order, transfer to another hospital before a first extubation attempt, received a tracheostomy before an extubation attempt (Supplemental Table 3 for the indications), and pregnant or post-partum women. The study protocol was approved by the Erasmus MC medical ethics committee (METC number 2022-0740). This study was performed under exception of consent, as a waiver was provided for research including COVID-19 patients (METC number 2020-0381). Patients with registered objections were exempted from this waiver. This study is reported according to the STROBE statement Checklists—STROBE (strobe-statement.org) (Supplemental Table 1).

### Data collection

During the COVID-19 pandemic, routine morning laboratory measurements were extended with additional biomarkers to help healthcare professionals easily detect complications during an infection with COVID-19. For this study, biomarkers measured on the day of extubation, patient characteristics and administration of medications (corticosteroids and IL-6 receptor antagonists (IL-6 RAs)), were extracted from electronic medical records. Cardiomyocyte injury was assessed in serum samples using Hs-TnT (ng/L), normal concentration < 14 ng/L. In addition, NT-proBNP (pmol/L) was measured as hemodynamic myocardial stress and heart failure biomarker, normal concentration < 15 pmol/L (corresponding with < 126 pg/mL). Inflammation was assessed in serum samples using IL-6 (pg/mL), normal concentration < 4.4 pg/ml, and PCT (ng/mL), normal concentration < 0.25 ng/mL. All biomarkers were measured on the Cobas 8000 analyzer (Roche Diagnostics, Mannheim, Germany).

### Definition of events

The primary endpoint was extubation failure, according to the WIND definition: the need for reintubation or death within the next seven days after extubation, regardless of whether post-extubation respiratory support was used or not [36]. Patients who did not experience extubation failure were defined as extubation success.

### Statistical analysis

Patient characteristics are presented as mean ± standard deviation or median [interquartile range, IQR], depending on the distribution of data. Categorical variables were described as frequency (percentage). Patients with extubation success and failure were compared using the Mann–Whitney U test for continuous variables and Pearson’s Chi-square test for categorical variables. Correlations of baseline characteristics with biomarker levels were also expressed using Pearson or Spearman correlation, depending on the distribution of data. Because of the highly skewed biomarker distributions, all biomarker values were log_2_ transformed for logistic regression analyses. Covariables with a p < 0.05 in univariable analyses were included in the multivariable logistic regression model. A multivariable logistic regression analysis was performed for the primary endpoint. The multivariable model was used with a minimum of ten endpoints per degree of freedom. Multicollinearity of highly correlated predictor variables was assessed using variance inflation factors. Furthermore, Receiver operating characteristics-curves were created for each biomarker separately and for the final model. In case of missing data, data was excluded from the analysis. Furthermore, additional analyses were performed with the most predictive cardiac (Hs-TnT) and inflammatory marker (PCT), based on the univariable analysis. Patients were classified based on a clinical cut-off for Hs-TnT and PCT value. Three groups were constructed. Group 1: elevated levels of Hs-TnT (≥ 14 ng/mL) and PCT (≥ 0.25 ng/mL). Group 2: elevated level of either Hs-TnT or PCT. Group 3: no increased values of Hs-TnT and PCT. Extubation failure rates were reported among the three groups. Furthermore, additional analyses were performed to evaluate the possible interaction between cardiac and inflammatory biomarkers. In patients with elevated and low Hs-TnT the odds ratio of log_2_ PCT was determined separately, and in patients with elevated and low PCT the odds ratio of log_2_ Hs-TnT was determined separately. Last, we evaluated a possible interaction between PCT and Hs-TnT by including an interaction term in the model.

Two-sided P values of < 0.05 were considered statistically significant. Data analysis was performed using R software for statistical computing version 4.2.1.

## Results

### Baseline characteristics

**Fig. 1:**
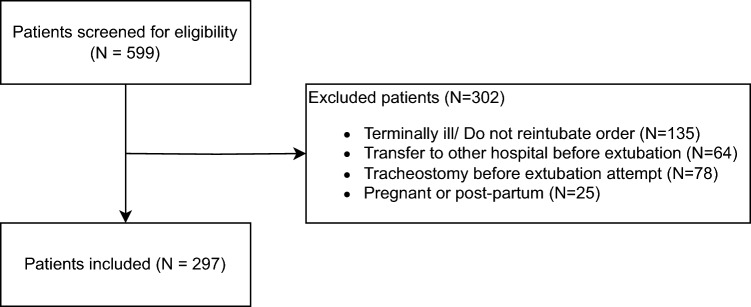
Flowchart of patient selection

Of the 599 screened patients between February 28th, 2020, and March 31st, 2022, 297 patients met the inclusion criteria (Fig. 1). Baseline characteristics are detailed in Table 1. The median age was 60 years [IQR, 51–67] and 70.0% were male. Patients with extubation failure were older, had a higher SOFA score on the day of extubation and a longer duration of mechanical ventilation before extubation. No difference in the Charlson Comorbidity Index (CCI) and comorbidities were observed between the success and failure groups. In Supplemental Table 4 all comorbidities according to the CCI are displayed. On the day of extubation NT-proBNP, Hs-TnT and PCT were higher in patients with extubation failure compared to patients with extubation success. For a few patients, biomarker values on the day of extubation could not be retrieved: 11 (3.7%) for NT-proBNP, 9 (3.0%) for Hs-TnT, 10 (3.4%) for PCT, 10 (3.4%) for IL-6.

**Table 1:** Baseline characteristics

	All patients (N = 297)	Extubation success (N = 233)	Extubation failure (N = 64)	P-value
Age (years)	60 [51–67]	59 [51–67]	64 [55–68]	0.03
Male sex, N (%)	208 (70.0)	162 (69.5)	46 (71.9)	0.85
BMI (kg/m^2^)	29.7 (± 5.8)	29.9 (± 5.7)	28.6 (± 5.8)	0.10
APACHE IV score	63 (± 39)	62 (± 43)	67 (± 20)	0.45
Charlson comorbidity index	2.0 [1.0–3.0]	2.0 [1.0–3.0]	3.0 [2.0–3.3]	0.06
Comorbidities, N (%)				
- Congestive heart failure	8 (2.7)	5 (2.1)	3 (4.7)	0.50
- Myocardial infarction	9 (3.0)	6 (2.6)	3 (4.7)	0.64
- PVD	10 (3.4)	8 (3.4)	2 (3.1)	1.00
- Hypertension	117 (39.4)	86 (36.9)	31 (48.4)	0.14
- Chronic Kidney disease	19 (6.4)	12 (5.2)	7 (10.9)	0.17
- Diabetes	81(27.2)	59 (25.3)	22 (34.3)	0.33
Administration of medications during ICU admission				
- IL-6 receptor antagonist	37 (31.4)*	33 (37.1)**	4 (13.8)***	0.14
- Corticosteroids	246 (82.8)	191 (82.0)	55 (85.9)	0.58
Duration of IMV before extubation (days)	9 [6–13]	8 [6–11]	11 [7–15]	0.001
SOFA score on the day of extubation	3 [2–5]	3 [2–4]	5 [3–7]	< 0.001
Total fluid balance until extubation (L)	0.9 [−1.0 – 3.0]	0.9 [−1.0 – 2.8]	1.0 [−0.3 – 3.3]	0.35
Fluid balance 24-h before extubation (L)	−0.4 [−1.0 to 0.2]	−0.4 [−1.1 to 0.2]	−0.3 [−0.9 to 0.2]	0.63
Biomarker levels on the day of extubation				
NT-proBNP (pmol/l)	27 [12–62]	25 [12–57]	44 [16–91]	0.01
Hs-TnT (ng/L)	14 [10–25]	13 [9–22]	20 [12–55]	< 0.001
Procalcitonin (ng/mL)	0.11 [0.06–0.22]	0.09 [0.06–0.17]	0.18 [0.10–0.39]	< 0.001
IL-6 (pg/mL)	74 [21–210]	78 [20–209]	68 [24–233]	0.91

Continuous variables are presented as mean ± standard deviation or median [interquartile range, IQR], as appropriate. Categorical variables are presented as N (%)

*BMI* Body Mass Index, *APACHE IV* Acute Physiology And Chronic Health Evaluation IV, *PVD* peripheral vascular disease, *IMV* Invasive Mechanical Ventilation, *SOFA* Sequential Organ Failure Assessment, *NT-proBNP* N-terminal pro-B-type natriuretic peptide, *Hs-TnT* High-sensitivity Troponin T, *PCT* Procalcitonin, *IL-6* Interleukin-6

^*^Data complete for 40% of all patients (n = 118), since 60% of patients was referred from another hospital, these data could not be retrieved

^**^Data complete for N = 89

^***^Data complete for N = 29

### Primary endpoint

Extubation failure occurred in 64 patients (21.5%). The reason for reintubation is specified in Supplemental Table 5. Death during ICU- or hospital stay occurred in 12 patients, resulting in an overall mortality of 4.0%. The mortality rate in patients with extubation failure was higher compared to patients with extubation success (15.6% vs. 0.9%, p < 0.001).

### Uni- and multivariable logistic regression model

In Table 2 the uni- and multivariable logistic regression analyses are presented. In univariable analysis log_2_ Hs-TnT, log_2_ NT-proBNP, and log_2_ PCT were associated with extubation failure. However, log_2_ IL-6 was not. In multivariable analyses log_2_ Hs-TnT was the only biomarker that was independently associated with extubation failure (adjusted OR, 1.38 (95% CI 1.02–1.90)). Furthermore, the duration of mechanical ventilation before extubation and SOFA score on the day of extubation were independently associated with extubation failure. No multicollinearity was found between all independent variables.

**Table 2:** Uni- and multivariable logistic regression model

Variable	Univariable OR (95% CI)	P-value	Multivariable OR (95% CI)	P-value
Age	1.02 [1.01–1.05]	0.04	1.02 [0.98–1.06]	0.34
Duration mechanical ventilation	1.09 [1.03–1.15]	0.001	1.06 [1.002–1.13]	0.04
SOFA score on day of extubation	1.42 [1.26–1.62]	< 0.001	1.32 [1.14–1.53]	< 0.001
Log_2_ NT-proBNP	1.24 [1.06–1.47]	0.01	0.92 [0.73–1.16]	0.41
Log_2_ Hs-TnT	1.72 [1.37–2.19]	< 0.001	1.38 [1.02–1.90]	0.04
Log_2_ PCT	1.38 [1.16–1.65]	0.001	1.13 [0.92–1.39]	0.21
Log_2_ IL-6	1.03 [0.91–1.16]	0.66	–	–

*OR* Odds Ratio, *SOFA* Sequential Organ Failure Assessment, *NT-proBNP* N-terminal pro-B-type natriuretic peptide, *Hs-TnT* High-sensitivity Troponin T, *PCT* Procalcitonin, *IL-6* Interleukin-6

Since all biomarkers were log_2_ transformed, one unit increase of the log₂-biomarker corresponds with a doubling of the biomarker’s value. Hence, odds ratios should be interpreted per doubling of the biomarker

**Fig. 2:**
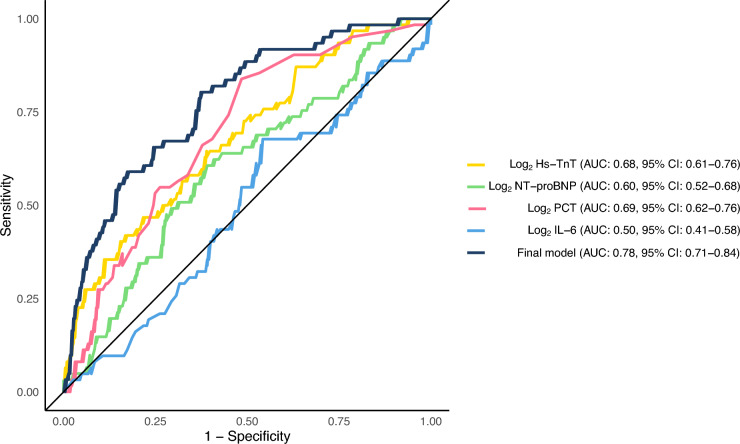
Receiver operating characteristics-curves showing the discriminative ability of each biomarker separately and the final model for the primary endpoint (extubation failure). *Hs-TnT* High-sensitivity Troponin T, *NT-proBNP* N-terminal pro-B-type natriuretic peptide, *PCT* Procalcitonin, *IL-6* Interleukin-6, *AUC* Area Under the Curve, *CI* Confidence Interval

In Fig. 2 the Receiver operating characteristics-curves are presented for all the biomarkers separately, and for the final model. The Area Under the Curve (AUC) for log_2_ Hs-TnT was 0.68 (95% CI 0.61–0.76), and the AUC for the final model was 0.78 (95% CI 0.71–0.84).

### Extubation failure in patients with elevated Hs-TnT and PCT

**Fig. 3:**
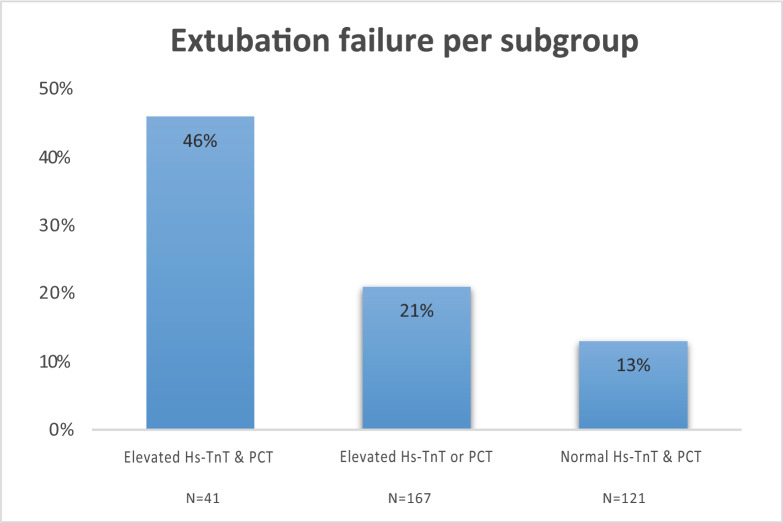
Extubation failure per subgroup. Group 1: elevated levels of Hs-TnT (≥ 14 ng/mL) and PCT (≥ 0.25 ng/mL). Group 2: elevated level of either Hs-TnT or PCT. Group 3: no elevated levels of Hs-TnT and PCT. *Hs-TnT* High-sensitivity Troponin T, *PCT* Procalcitonin

Patients were divided into three subgroups (based on clinical cut-offs): elevated Hs-TnT and PCT, elevated Hs-TnT or PCT, normal Hs-TnT and PCT. In Fig. 3 the percentages of extubation failure among the three groups are displayed. In patients with both elevated Hs-TnT and PCT values 46% (19/41) experienced extubation failure, while in patients with both normal Hs-TnT and PCT 13% (16/121) experienced extubation failure. Since extubation failure occurred more frequently in patients with elevated Hs-TnT and PCT, we investigated whether there was a potential interaction between these biomarkers in relation to extubation failure (i.e., patients with more inflammation have a stronger association between Hs-TnT and extubation failure or vice versa). This was performed by an additional analysis of the prognostic value of log_2_ PCT in subgroups of patients with Hs-TnT < 14 and ≥ 14 ng/L; and of the prognostic value of log_2_ Hs-TnT in subgroups of patients with PCT < 0.25 ng/mL and ≥ 0.25 ng/mL. There were no significant differences between the different subgroups based on biomarker values, as presented in Supplemental Fig. 1. Moreover, in logistic regression analysis an interaction term between Hs-TnT and PCT was not significantly predictive of extubation failure (p = 0.58).

### Correlation between biomarkers and baseline characteristics

To understand which characteristics were associated with elevated biomarker levels, we calculated the correlation coefficients between biomarkers and baseline variables (Table 3). Overall, correlations were weak to moderate since there were no correlations above > 0.50. Notably, cardiac biomarkers were not strongly correlated with cardiovascular comorbidities (congestive heart failure, myocardial infarction). However, there was a significant, but moderate correlation for both NT-proBNP and Hs-TnT with the Charlson Comorbidity Index (0.43 and 0.35, respectively) and with APACHE IV score. Furthermore, the correlation between Hs-TnT and duration of mechanical ventilation and SOFA score was stronger in comparison to other characteristics. NT-proBNP was not significantly related to the total fluid balance before extubation and the fluid balance 24-h before extubation.

**Table 3:** Correlation coefficients *(r)* of NT-proBNP, Hs-TnT, PCT and IL-6 with clinical characteristics

	NT-proBNP *(r)*	Hs-TnT *(r)*	PCT *(r)*	IL-6 *(r)*
Clinical characteristics				
Age	0.345**	0.279**	0.168**	−0.015
Sex, male	0.025	−0.043	0.034	−0.091
BMI	−0.076	−0.011	−0.110	−0.038
Charlson comorbidity index	0.435**	0.352**	0.267**	0.019
Hypertension	0.185**	0.215**	0.104	−0.072
Chronic kidney disease	0.263**	0.226**	0.147*	0.074
Congestive heart failure	0.157**	0.060	0.057	0.005
Myocardial infarction	0.011	−0.025	0.014	−0.076
Peripheral vascular disease	0.101	0.155**	0.026	0.003
Diabetes mellitus	0.200**	0.148*	0.230**	0.011
IL-6 receptor antagonist use	−0.123	−0.038	−0.054	0.017
Corticosteroids use	0.145	0.081	−0.065	0.047
Duration of mechanical ventilation	0.128*	0.301**	0.199**	−0.113
APACHE IV score	0.400**	0.354**	0.141	0.057
SOFA score on day of extubation	0.253**	0.329**	0.288**	−0.052
Total fluid balance until extubation	0.063	−0.020	0.093	−0.012
Fluid balance 24 h before extubation	−0.080	−0.008	0.145*	−0.066

*BMI* Body Mass Index, *APACHE IV* Acute Physiology And Chronic Health Evaluation IV, *SOFA* Sequential Organ Failure Assessment, *NT-proBNP* N-terminal pro-B-type natriuretic peptide, Hs-TnT, High-sensitivity Troponin T; PCT, Procalcitonin, IL-6, Interleukin-6

Correlations in bold correspond with a correlation above 0.30

^*^p < 0.05; ** p < 0.01

Correlations between clinical characteristics and inflammatory markers (PCT and IL-6) were weak, since none of the correlations was higher than 0.30. Furthermore, there was no correlation between IL-6 and IL-6 RA use and no correlation between PCT and corticosteroid use.

## Discussion

To our knowledge, this is the first study investigating the association of both cardiac and inflammatory biomarkers in relation to extubation failure in C-ARDS patients. We found that Hs-TnT, NT-proBNP and PCT measured on the day of extubation were significantly associated with extubation failure. After multivariable adjustment for clinical variables (age, duration of mechanical ventilation, SOFA score), Hs-TnT was the only biomarker that was independently associated with extubation failure. Although patients with both elevated Hs-TnT (≥ 14 ng/mL) and elevated PCT (≥ 0.25 ng/mL) carried the highest risk of extubation failure (46%), there was no interaction effect between Hs-TnT and PCT in relation to extubation failure.

### Cardiac biomarkers

Previous research in patients with C-ARDS mainly investigated the role of cardiac biomarkers in relation to (in-hospital) mortality rather than extubation failure [12, 13, 37], demonstrating that both NT-proBNP and Hs-TnT were associated with mortality. In these studies, cardiac biomarkers were measured earlier in the course of the disease (first 24 h of admission or peak measurement [12, 13, 37]). In contrast to previous literature [33], in our study, NT-proBNP was not independently associated with extubation failure. In our study, both total fluid balance and fluid balance 24-h before extubation were negative. We hypothesized that the association between NT-proBNP and extubation failure did not remain, because fluids had been withdrawn very strictly before proceeding with extubation. This is supported by the absence of a strong correlation between fluid balance and extubation failure.

Recently, Ionescu et al. [32] reported an association between peak troponin level and extubation failure in patients with COVID-19. We found that Hs-TnT measured on the day of extubation is associated with extubation failure. A biomarker measurement taken closer to the moment of extubation may be more clinically relevant than a peak measurement during ICU admission, which could reflect, for example, an episode of severe hypoxemia with type II ischemia, regardless of coronary status. Patients in our cohort frequently had cardiovascular risk factors such as diabetes mellitus, hypertension, and high BMI, but rarely a history of congestive heart failure or myocardial infarction. It is most plausible that patients had occult coronary artery disease and, during their work-up for extubation, experienced a mismatch in myocardial oxygen supply and demand, leading to cardiomyocyte injury and troponin release. This imbalance may also contribute to the occurrence of extubation failure. Patients with classical ARDS often have cardiovascular risk factors as well [38], which suggests that these findings may also be applicable to them.

### Inflammatory biomarkers

IL-6 is a proinflammatory cytokine that is involved in multiple infectious diseases and can also exhibit anti-inflammatory effects [39]. In patients with COVID-19, higher levels of IL-6 were associated with more severe inflammation and mortality [40]. Moreover, in patients with non-C-ARDS higher IL-6 levels were related to extubation failure [18]. However, these levels were measured on day 3 after start of mechanical ventilation. In our study the median duration of mechanical ventilation was 9 days, which could explain why we did not find an association between IL-6 and extubation failure. Furthermore, administration of IL-6 RAs (Tocilizumab or Sarilumab) might have altered the level of IL-6. Since 60% of our cohort was originally admitted in other hospitals and were then transferred to our hospital due to ICU capacity strain, data on administering of tocilizumab was largely unknown*.* However, the administration of an IL-6 RA was implemented in January 2021, after the preprint of the publication of the REMAP-CAP and RECOVERY trial [41, 42]. Hence, we would expect that almost 60% (percentage of patients admitted after January 2021) of the cohort did receive IL-6 RAs. IL-6 RAs can lead to an initial increase in circulating IL-6, but generally lead to a reduction in cytokine and acute phase reactant production [43], which might contribute to the absent association between IL-6 and extubation failure in this study. Similar to IL-6 RAs, corticosteroids were also part of the treatment protocol in C-ARDS and might have had effect on the inflammatory response and connected IL-6 (and PCT) dynamics. PCT is a prohormone of calcitonin and acts as an acute-phase protein during systemic inflammatory reactions [44]. Procalcitonin is mostly known to assess the risk of secondary bacterial infection [45, 46]. However, in patients with an ongoing COVID-19 infection PCT can remain elevated [47–50]. Furthermore, PCT remained associated with disease severity in C-ARDS even after correction for bacterial co-infection [51]. To date, no previous reports have been published on the association between PCT and extubation failure. In our study we found that unadjusted levels of PCT on the day of extubation were higher in patients with extubation failure.

### Effect of elevated cardiac and inflammatory biomarkers

Given the higher rates of extubation failure in patients with elevated Hs-TnT and PCT, we determined whether patients with more inflammation have a stronger association between Hs-TnT and extubation failure and vice versa. We observed an unfavorable additive effect on extubation failure of having both elevated Hs-TnT and PCT without an interaction effect between cardiac and inflammatory markers, highlighting that patients with both troponin release and ongoing inflammation are at increased risk for extubation failure.

Besides well-known markers, there has been attention for other biomarkers in relation to extubation failure [52]. Carbohydrate-antigen (CA-125) has been shown to be a promising surrogate of congestion and inflammation in patients with heart failure [53]. Lombuli et al. showed a positive association between CA-125 and post-extubation respiratory failure, suggesting a potential role for CA-125 in identifying cardiac dysfunction as cause of weaning failure [52].

### Strengths and limitations

Our study has several strengths. First, the sample size for an ICU population is relatively large. Second, despite the retrospective study design, missing data on biomarker measurements was low (< 3.7%). Third, this is the first study focusing on two pathways (cardiac and inflammatory) in relation to extubation failure. However, our study has some limitations. First, this was a retrospective study, reflecting extubation practice in a single center in patients with C-ARDS. Due to the study design, factors that are potentially relevant in extubation failure were not available (e.g., MRC sum score, cough strength, respiratory secretions), and not all common inflammatory markers (e.g., CRP) could be retrieved. Future studies are needed to validate our findings in patients with ARDS not caused by COVID-19, as differences in biomarkers (e.g., endothelial and epithelial pulmonary injury) between classical ARDS and C-ARDS have been reported [54]. Second, apart from cardiac and inflammatory pathways, also other pathways may be relevant in the context of extubation failure. For example, biomarkers on respiratory muscle work (e.g. creatinine kinase muscle type and fast/slow sTnI) have potential in patients weaning from mechanical ventilation [55]. Third, since biomarker measurements were part of the standard clinical care, physicians were not blinded. Abnormal biomarker values might have altered physicians’ decisions and potentially biased our findings. Finally, we were not able to retrieve all data on administration of IL-6 RAs, since patients were transferred to our ICU from the whole country, and patients were likely to have received an IL-6 RA elsewhere. However, since there was no difference in administration of the medication between patients with extubation failure and success and there was no correlation between IL-6 and IL-6 RA use, it is unlikely that the absence of these data has affected our results.

### Future perspectives

Future studies should focus on an integrative approach of biomarkers combined with relevant clinical factors to predict extubation failure. We would suggest measuring biomarkers in patients who are mechanically ventilated for more than 48 h, who meet ready to wean criteria, and are subjected to an SBT. Incorporating biomarkers alongside ready to wean criteria may enhance the decision-making process for weaning from mechanical ventilation. This could especially be useful for patients with high risk of extubation failure (e.g., > 65 years, underlying chronic cardiac or respiratory disease [56], first failed SBT [57]). Furthermore, future studies should also focus on an assessment of biomarkers on multiple time points, for instance before and after the SBT. This enables a better understanding of the trajectory of the biomarker and allows us to investigate whether the trend of the biomarkers can assist physicians in successfully weaning patients from mechanical ventilation. In addition, these investigations could serve as a steppingstone to optimize treatment for patients with failed extubation attempts. By initiating personalized biomarker panels, the underlying diagnosis of extubation failure can be clarified more quickly, leading to optimization of treatment strategies.

## Conclusions

This study shows that cardiac and inflammatory biomarkers (Hs-TnT, NT-proBNP and PCT) measured on the day of extubation are associated with extubation failure in mechanically ventilated patients with C-ARDS. Patients with both elevated Hs-TnT and PCT were at the highest risk of extubation failure. Since Hs-TnT measured on the day of extubation is independently associated with extubation failure, Hs-TnT could offer additional objective measures for assessing readiness for extubation.

## Supplementary Information

Supplementary material 1.

## Data Availability

The datasets used during the current study are available from the corresponding author on reasonable request.

## Abbreviations

APACHE IV: Acute physiology and chronic health evaluation IV
ARDS: Acute respiratory distress syndrome
AUC: Area under the curve
C-ARDS: COVID-19 acute respiratory distress syndrome
CCI: Charlson comorbidity index
COPD: Chronic obstructive pulmonary disease
IL-6: Interleukin-6
IL-6 RA: Interleukin-6 receptor antagonist
IMV: Invasive mechanical ventilation
Hs-TnT: High-sensitivity Troponin T
NT-proBNP: N-terminal pro–B-type natriuretic peptide
PCT: Procalcitonin
PVD: Peripheral vascular disease
SBT: Spontaneous breathing trial
SOFA: Sequential Organ Failure Assessment
STROBE: Strengthening the reporting of observational studies in epidemiology
WIND: Weaning according to a new definition
