# Supplementary material for: The prognostic role of cardiac and inflammatory biomarkers in extubation failure in patients with COVID-19 acute respiratory distress syndrome
Source: Ann Intensive Care. 2025 Jan 9;15:2. doi: 10.1186/s13613-025-01425-3 (PMC11711961; doi:10.1186/s13613-025-01425-3)
Supplement: Supplementary file 1 — Supplementary material 1. [file 13613_2025_1425_MOESM1_ESM.docx]

**Supplemental files**

**Supplemental Table 1 - STROBE Statement** —Checklist of items that should be included in reports of *cohort studies*

|  | Item No | Recommendation | Page No |
| --- | --- | --- | --- |
| **Title and abstract** | 1 | (*a*) Indicate the study’s design with a commonly used term in the title or the abstract | 1 - 2 |
|  |  | (*b*) Provide in the abstract an informative and balanced summary of what was done and what was found | 2 |
| Introduction | | | |
| Background/rationale | 2 | Explain the scientific background and rationale for the investigation being reported | 4 |
| Objectives | 3 | State specific objectives, including any prespecified hypotheses | 4 - 5 |
| Methods | | | |
| Study design | 4 | Present key elements of study design early in the paper | 6 |
| Setting | 5 | Describe the setting, locations, and relevant dates, including periods of recruitment, exposure, follow-up, and data collection | 6 - 7 |
| Participants | 6 | (*a*) Give the eligibility criteria, and the sources and methods of selection of participants. Describe methods of follow-up | 6 - 7 |
|  |  | (*b*) For matched studies, give matching criteria and number of exposed and unexposed |  |
| Variables | 7 | Clearly define all outcomes, exposures, predictors, potential confounders, and effect modifiers. Give diagnostic criteria, if applicable | 6 - 8 |
| Data sources/ measurement | 8* | For each variable of interest, give sources of data and details of methods of assessment (measurement). Describe comparability of assessment methods if there is more than one group | 6 - 8 |
| Bias | 9 | Describe any efforts to address potential sources of bias | 6 - 8 |
| Study size | 10 | Explain how the study size was arrived at | 6 - 9 |
| Quantitative variables | 11 | Explain how quantitative variables were handled in the analyses. If applicable, describe which groupings were chosen and why | 6 - 8 |
| Statistical methods | 12 | (*a*) Describe all statistical methods, including those used to control for confounding |  |
|  |  | (*b*) Describe any methods used to examine subgroups and interactions | 7 - 9 |
|  |  | (*c*) Explain how missing data were addressed |  |
|  |  | (*d*) If applicable, explain how loss to follow-up was addressed |  |
|  |  | (*e*) Describe any sensitivity analyses |  |
| Results | | |  |
| Participants | 13* | (a) Report numbers of individuals at each stage of study—eg numbers potentially eligible, examined for eligibility, confirmed eligible, included in the study, completing follow-up, and analysed | 9 |
|  |  | (b) Give reasons for non-participation at each stage | 9 |
|  |  | (c) Consider use of a flow diagram | 9 |
| Descriptive data | 14* | (a) Give characteristics of study participants (eg demographic, clinical, social) and information on exposures and potential confounders | 9 |
|  |  | (b) Indicate number of participants with missing data for each variable of interest | 9 |
|  |  | (c) Summarise follow-up time (eg, average and total amount) | - |
| Outcome data | 15* | Report numbers of outcome events or summary measures over time | 9 |

| Main results | 16 | (*a*) Give unadjusted estimates and, if applicable, confounder-adjusted estimates and their precision (eg, 95% confidence interval). Make clear which confounders were adjusted for and why they were included | 9 - 11 |
| --- | --- | --- | --- |
|  |  | (*b*) Report category boundaries when continuous variables were categorized | 8 |
|  |  | (*c*) If relevant, consider translating estimates of relative risk into absolute risk for a meaningful time period | - |
| Other analyses | 17 | Report other analyses done—eg analyses of subgroups and interactions, and sensitivity analyses | 10 - 11 |
| Discussion | | | |
| Key results | 18 | Summarise key results with reference to study objectives | 12 |
| Limitations | 19 | Discuss limitations of the study, taking into account sources of potential bias or imprecision. Discuss both direction and magnitude of any potential bias | 15 - 16 |
| Interpretation | 20 | Give a cautious overall interpretation of results considering objectives, limitations, multiplicity of analyses, results from similar studies, and other relevant evidence | 16 - 17 |
| Generalisability | 21 | Discuss the generalisability (external validity) of the study results | 15 |
| Other information | | | |
| Funding | 22 | Give the source of funding and the role of the funders for the present study and, if applicable, for the original study on which the present article is based | 18 |

*Give information separately for exposed and unexposed groups.

**Note:** An Explanation and Elaboration article discusses each checklist item and gives methodological background and published examples of transparent reporting. The STROBE checklist is best used in conjunction with this article (freely available on the Web sites of PLoS Medicine at http://www.plosmedicine.org/, Annals of Internal Medicine at http://www.annals.org/, and Epidemiology at http://www.epidem.com/). Information on the STROBE Initiative is available at http://www.strobe-statement.org.

**Table S2. Ready to wean criteria**

| **Ready to wean criteria** |
| --- |
| SaO2 > 90% with FiO2 ≤ 40% |
| PEEP < 10 cm H_2_O |
| Respiratory Rate ≤ 35 /minute |
| Tidal volume >5 ml/kg |
| RSBI < 105 |
| No respiratory acidosis |
| HR ≤ <140 b/m |
| Systolic Blood Pressure 90 -160 mmHg |

PEEP; Positive End Expiratory Pressure, RSBI; Rapid Shallow Breathing Index, HR; Heart Rate,

Ta**ble S3. Indications tracheostomy**

| **Indication for tracheostomy** |
| --- |
| Expected prolonged mechanical ventilation |
| Weaning failure (critical illness poly neuropathy) |
| Neurologic patients with GCS <7-9 and/or reduced swallow and cough reflex |
| Severely compromised lung function before ICU admission |
| Upper airway obstruction |
| Copious airway secretions/ sputum retention |

GCS; Glasgow Coma Scale

**Table S4. Additional baseline characteristics**

|  | All patients (N=297) | Extubation success (N=233) | Extubation failure  (N=64) | P-value |
| --- | --- | --- | --- | --- |
| Origin admission   - Emergency department EMC - Emergency department (referral hospital) - Hospital ward EMC - Hospital ward (referral hospital) - Intensive Care (referral hospital) | 27 (9.4)  3 (1.0)  91 (30.5)  8 (2.7)  168 (56.4) | 21 (9.0)  3 (1.3)  68 (29.2)  8 (3.4)  133 (57.1) | 6 (9.4)  0  23 (35.9)  0  35 (54.7) |  |
| Number of comorbidities according to the Charlson Comorbidity index  0  1  2  3  4  5  6  7  8  9  10 | 51 (17.1)  49 (16.4)  62 (20.8)  68 (22.9)  36 (12.1)  17 (5.7)  6 (2.0)  3 (1.0)  2 (0.7)  1 (0.3)  2 (0.7) | 43 (18.4)  44 (18.8)  45 (19.2)  50 (21.4)  30 (12.9)  11 (4.7)  4 (1.7)  3 (1.3)  0 (0.0)  1 (0.4)  2 (0.9) | 8 (12.5)  5 (7.8)  17 (26.6)  18 (28.1)  6 (7.8)  6 (9.4)  2 (3.1)  0 (0.0)  2 (3.1)  0 (0.0)  0 (0.0) | 0.032 |
| Age   - < 50 Years - 50 - 50 years - 60 - 69 years - 70 – 79 years - > 80 years | 66 (22.2)  80 (26.9)  96 (32.3)  53 (17.8  2 (0.7) | 55 (23.6)  67 (28.8)  71 (30.5)  38 (16.3)  2 (0.9) | 11 (17.2)  13 (20.3)  25 (39.1)  15 (23.4)  0 | 0.25 |
| Diabetes Mellitus   - None - Uncomplicated - End organ damage | 216 (72.7)  72 (24.2)  9 (3.0) | 174 (74.7)  52 (22.3)  7 (3.0) | 42 (65.6)  20 (31.2)  2 (3.1) | 0.329 |
| Liver Disease   - None - Mild - Moderate - Severe | 288 (97.0)  8 (2.7)  1 (0.3) | 225 (96.6)  8 (3.4)  0 | 63 (98.4)  0  1 | 0.053 |
| Malignancy   - None - Any leukemia, localized or solid tumor - Metastatic solid tumor | 275 (92.6)  17 (5.7)  5 (1.7) | 216 (92.7)  12 (5.2)  5 (2.1) | 59 (92.2)  5 (7.8)  0 | 0.369 |
| Aids | 1 (0.3) | 1 (0.4) | 0 | 1.00 |
| Moderate to severe chronic kidney disease | 19 (6.4) | 12 (5.2) | 7 (10.9) | 0.165 |
| Congestive heart failure | 8 (2.7) | 5 (2.1) | 3 (4.7) | 0.499 |
| Myocardial infarction | 9 (3.0) | 6 (2.6) | 3 (4.7) | 0.644 |
| COPD | 14 (4.7) | 13 (5.6) | 1 (1.6) | 0.312 |
| Peripheral vascular disease | 10 (3.4) | 8 (3.4) | 2 (3.1) | 1.00 |
| CVA | 10 (3.4) | 7 (3.0) | 3 (4.7) | 0.787 |
| Dementia | 1 (0.3) | 0 | 1 (1.6) | 0.488 |

**Abbreviations:** EMC, Erasmus Medical Center, COPD, Chronic Obstructive Pulmonary Disease, CVA, Cerebrovascular Accident.

**Table S5 - Reason for extubation failure**

| **Reason for extubation failure, n (%)** | **Extubation failure (N=64)** |
| --- | --- |
| 1. Upper airway obstruction (stridor)  2. Aspiration or excess of pulmonary secretions  3. Respiratory failure (increased WOB, desaturation, tachypnea)  4. Congestive heart failure (cardiopulmonary oedema)  5. Encephalopathy (resulting in hypoventilation and increased PaCO_2_)  6. Other (i.e., hemodynamic instability because of major bleed) | 5 (7.8)  11 (17.2)  45 (70.3)  0  2 (3.1)  1 (1.6) |

Abbreviations: WOB, work of breathing, PaCO_2,_ Partial pressure of arterial carbon dioxide


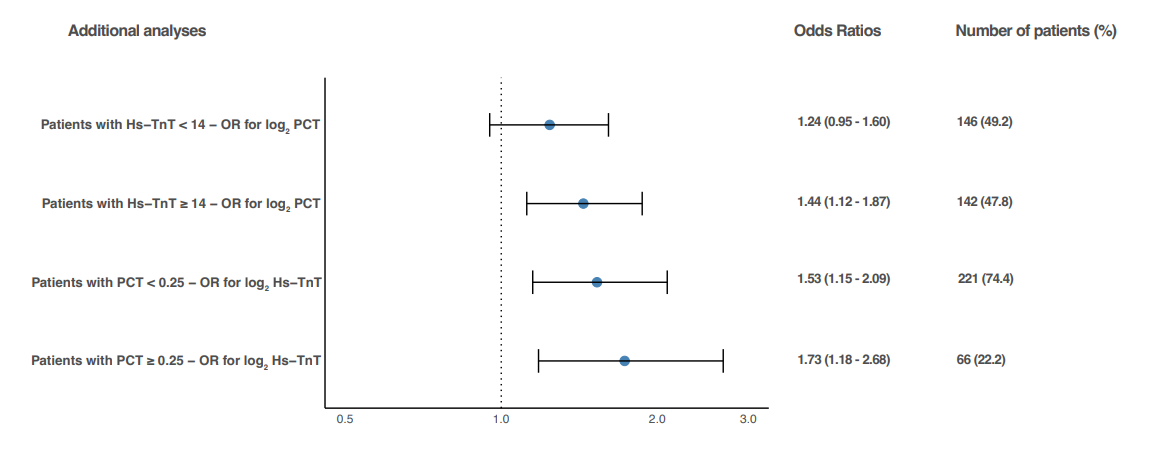


**Figure S1 Additional analyses for prognostic value of log_2_ PCT and log_2_ Hs-TnT**

Additional analysis of the prognostic value of log­_2_ PCT in subgroups of patients with Hs-TnT <14 and ≥14 ng/L; and of the prognostic value of log_2_ Hs-TnT in subgroups of patients with PCT <0.25 ng/mL and ≥0.25 ng/mL
